# Acute stress causes rapid synaptic insertion of Ca^2+^-permeable AMPA receptors to facilitate long-term potentiation in the hippocampus

**DOI:** 10.1093/brain/awt293

**Published:** 2013-12-10

**Authors:** Garry Whitehead, Jihoon Jo, Ellen L. Hogg, Thomas Piers, Dong-Hyun Kim, Gillian Seaton, Heon Seok, Gilles Bru-Mercier, Gi Hoon Son, Philip Regan, Lars Hildebrandt, Eleanor Waite, Byeong-Chae Kim, Talitha L. Kerrigan, Kyungjin Kim, Daniel J. Whitcomb, Graham L. Collingridge, Stafford L. Lightman, Kwangwook Cho

**Affiliations:** 1 Henry Wellcome Laboratories for Integrative Neuroscience and Endocrinology, School of Clinical Sciences, Faculty of Medicine and Dentistry, University of Bristol, Whitson Street, Bristol BS1 3NY, UK; 2 Chonnam-Bristol Frontier Laboratory, Biomedical Research Institute, Chonnam National University Hospital, Jebong-ro, Gwangju 501-757, Republic of Korea; 3 Department of Biomedical Engineering, Jungwon University, 85 Munmu-ro, Goesan-gun, Chungcheongbuk-do 367-805, Republic of Korea; 4 Department of Biological Sciences and 21st Frontier Program in Neuroscience, Seoul National University, Seoul 151-742, Republic of Korea; 5 Centre for Synaptic Plasticity, University of Bristol, Bristol, UK; 6 School of Physiology and Pharmacology, University of Bristol, UK; 7 Department of Neurology, Chonnam National University Medical School, Gwangju, Republic of Korea; 8 Department of Brain and Cognitive Sciences, Seoul National University, Republic of Korea

**Keywords:** long-term potentiation, metaplasticity, glucocorticoids, glutamate receptor, calcium

## Abstract

The neuroendocrine response to episodes of acute stress is crucial for survival whereas the prolonged response to chronic stress can be detrimental. Learning and memory are particularly susceptible to stress with cognitive deficits being well characterized consequences of chronic stress. Although there is good evidence that acute stress can enhance cognitive performance, the mechanism(s) for this are unclear. We find that hippocampal slices, either prepared from rats following 30 min restraint stress or directly exposed to glucocorticoids, exhibit an *N*-methyl-d-aspartic acid receptor-independent form of long-term potentiation. We demonstrate that the mechanism involves an NMDA receptor and PKA-dependent insertion of Ca^2+^-permeable AMPA receptors into synapses. These then trigger the additional NMDA receptor-independent form of LTP during high frequency stimulation.

## Introduction

Chronic stress is well recognized to be an important risk factor for both depression and memory impairment (McEwen *et al.*, 2012) and a recent study in over 11 000 individuals from the Swedish Twin Registry showed that chronic morbidity is associated with significant impairment of cognitive function (Caracciolo *et al.*, 2013). In experimental studies, stress has been specifically associated with effects on cognition (Kim and Diamond, 2002; de Kloet *et al.*, 2005; Roozendaal *et al.*, 2010) and memory processing (de Quervain *et al.*, 2009). Deleterious effects are most apparent when stress has been prolonged, whereas in contrast the acute response to a stressor is adaptive with increased attention, vigilance and improved cognitive performance (de Kloet *et al.*, 1999; Joels *et al.*, 2008, 2011; Sandi, 2011). These and many other studies clearly show that in response to stress the brain exhibits both structural and functional plasticity, and it is this capacity that in part provides us with an opportunity to develop novel pharmacological strategies for the treatment of a wide range of clinical conditions from dementia and depression to epilepsy and stroke.

The first stage in the pathway to translating the positive effects of the acute stress response is to establish the mechanism(s) through which the stressor enhances synaptic plasticity, and in particular how the stress hormones cortisol (in human) and corticosterone (in the rodent) alter synaptic function. Previous studies suggest that glucocorticoids rapidly modulate excitatory synaptic transmission (Karst *et al.*, 2005), at least in part through their regulation of glutamate receptors (Groc *et al.*, 2008; Yuen *et al.*, 2011). As excitatory glutamate receptors are critically involved in long-term synaptic plasticity and learning and memory (Bliss and Collingridge, 1993; Neves *et al.*, 2008), the mechanisms underlying the glucocorticoid regulation of glutamatergic synaptic plasticity might provide links between glucocorticoids and the modulation of memory processes during stress.

The α-amino-3-hydroxy-5-methylisoxazole-4-propionic acid receptor (AMPAR) is a major glutamatergic receptor involved in excitatory synaptic transmission. The trafficking of AMPARs to the synapse is widely accepted to be critical in long-term synaptic plasticity (Isaac *et al.*, 1995; Hayashi *et al.*, 2000; Kessels and Malinow, 2009), a process thought to underlie learning and memory (Bliss and Collingridge, 1993; Lamprecht and LeDoux, 2004; Neves *et al.*, 2008; Ho *et al.*, 2011). Both the number and subunit composition of postsynaptic AMPARs are able to determine the activity-dependent changes responsible for long-term potentiation (LTP) (Malinow and Malenka, 2002; Bredt and Nicoll, 2003; Sheng and Hyoung Lee, 2003). In particular, LTP involves the insertion of AMPARs into the synaptic region and a concurrent increase in AMPAR-mediated transmission (Kessels and Malinow, 2009). In addition to changes in synaptic receptor number during synaptic events, changes in AMPAR subunit composition can also be a fundamental process in regulating synaptic strength (Liu and Cull-Candy, 2000; Cull-Candy *et al.*, 2006; Liu and Zukin, 2007). Such events occur in response to stress; exposure to stressors has been shown to mediate the synaptic insertion of GluA2-lacking, Ca^2+^-permeable AMPARs (CP-AMPARs) (Clem and Huganir, 2010; Savtchouk and Liu, 2011). However, the mechanism through which stress modifies AMPAR composition, and the consequences of this for synaptic plasticity, are unknown.

We hypothesized that acute stress or transient exposure to glucocorticoids would enhance mechanisms underlying synaptic efficacy. In the present study, we have characterized the effects of acute stress and glucocorticoid exposure on the magnitude and induction mechanism of hippocampal LTP using *ex vivo* and *in vitro* models. We find that acute stressors elicit an *N*-methyl-d-aspartate receptor (NMDAR)-dependent type of metaplasticity that enhances LTP through the priming of a form of LTP that is independent of, but additive with, NMDAR-dependent LTP. This stress-induced LTP is independent of protein synthesis but is associated with activation of protein kinase A (PKA), phosphorylation of S845 of GluA1, and insertion of GluA1 subunits into the plasma membrane. Stress-induced LTP requires Ca^2+^ for its induction and is blocked by IEM-1460 (IEM), an inhibitor of CP-AMPARs. Thus at CA1 synapses in the hippocampus, conventional NMDAR-LTP can coexist with a distinct form of LTP that is primed by stress and involves CP-AMPARs in its induction.

## Materials and methods

### Animals

Four to five-week old male Wistar rats were received from Charles River. They were housed in small groups with free access to water and food. They were subjected to a 12 h light/12 h dark cycle with the light phase commencing at 8.00 am. Animals were sacrificed between 10:00 am and 11:00 am by cervical dislocation in accordance with the UK Animals Scientific Procedures Act of 1986.

### Restraint stress

Rats were physically restrained in 50 ml Falcon tubes for 30 min without food or water. Control rats were housed in their usual cages under normal conditions. Animals were sacrificed immediately following restraint stress by decapitation.

### Slice preparation

The brain was quickly removed and transferred to ice-cold artificial CSF containing: 124 mM NaCl, 3 mM KCl, 26 mM NaHCO_3_, 1.25 mM NH_2_PO_4_, 2 mM CaCl_2_, 1 mM MgSO_4_, and 10 mM glucose. A mid-sagittal cut was made in the brain and one hemisphere was placed back into the ice-cold artificial CSF until it was required. Transverse hippocampal slices (400 µm) were cut using a Mcllwain tissue chopper (Mickle Laboratory Engineering Co. Ltd.) and allowed to stabilize in artificial CSF for 1 h while constantly perfused in 95% O_2_ / 5% CO_2_ mixture.

### Electrophysiology

A recovery period, of approximately 60 min, was allowed for the tissue to recover from the slicing procedure and for stable responses to be obtained. Extracellular field potentials were recorded in the CA1 region using glass electrodes containing NaCl (3 M). Stimulating electrodes were placed in the subiculum and CA2 (Schaffer collateral pathway). Stimuli were delivered alternately to the two electrodes (each electrode 0.016 Hz). LTP was evoked by two trains of tetanus stimuli (each 100 Hz, 1 s; repeated after a 30 s interval). The slope of the evoked field potential responses was measured and expressed relative to the normalized preconditioning baseline. Data were captured and analysed using WinLTP (www.winltp.com). Experiments in which changes in the fibre volley occurred were discarded.

### Biotinylation and NeutrAvidin pull-down

Surface biotinylation of acute slices was performed as described previously with some modifications (Thomas-Crusells *et al.*, 2003). Briefly, slices were initially washed twice in artificial CSF and subsequently incubated in artificial CSF containing 1 mg/ml Sulfo-NHS-SS-Biotin for 45 min at 4°C to allow for labelling of all surface membrane proteins. Biotinylated tissue was then homogenized in lysis buffer containing 25 mM Tris (pH 7.6), 150 mM NaCl, 1% Triton™ X-100, 0.5% sodium deoxycholate, 0.1% SDS, 1 mM NaF and a cocktail of protease inhibitors (Sigma). The lysate was centrifuged at 21 000*g* to remove nuclei and cellular debris. Total protein concentration was determined using the BCA assay (Pierce). A small amount of the lysate was removed for whole-cell analysis later. Subsequently, 100 µl of StreptaAvidin beads (Thermo Scientific Inc) were added to 500 µg of protein lysate and placed on a rotator at 4°C for 2 h. Samples were then washed five times in wash buffer (25 mM Tris pH 7.6, 150 nM NaCl, 0.5% Triton™ X-100); beads were pulled-down after each wash by gentle centrifugation. Bound proteins were eluted in 2× SDS reducing buffer and gently heated at 60°C for 30 min. The resulting supernatant was transferred to new tubes and heated at 90°C for 5 min before gel loading.

### Western blot and data analysis

Proteins suspended in Laemmli buffer were separated using 10% SDS-PAGE. Subsequently, the proteins were transferred onto PVDF membrane (Bio-Rad) and incubated with the relevant primary antibodies. The following polyclonal antibody was used: anti-panCadherin (1/1000) from Cell Signalling. Monoclonal antibodies used include: anti-β-actin (1/10 000) from Abcam; anti-phospho GluR1 S845 (1/2,000) from Millipore; anti-GluR1 (1/250 dilution) from Santa Cruz; and anti-GluR2 (1/1000) from Chemicon. Membranes were then incubated in either rabbit or mouse IgG antibodies (1/5000 dilution, Millipore) conjugated to horseradish peroxidise and immunoblotted using the ECL detection system (Thermo Scientific Inc.). Optical densities of immune reactive bands were measured using ImageJ software (NIH) and statistical analysis conducted with SigmaPlot (Systat Software, Inc., USA). The statistical significance of the data was analysed by Students *t*-test and a probability level of *P < *0.05 was considered statistically significant.

## Results

**Figure 1:**
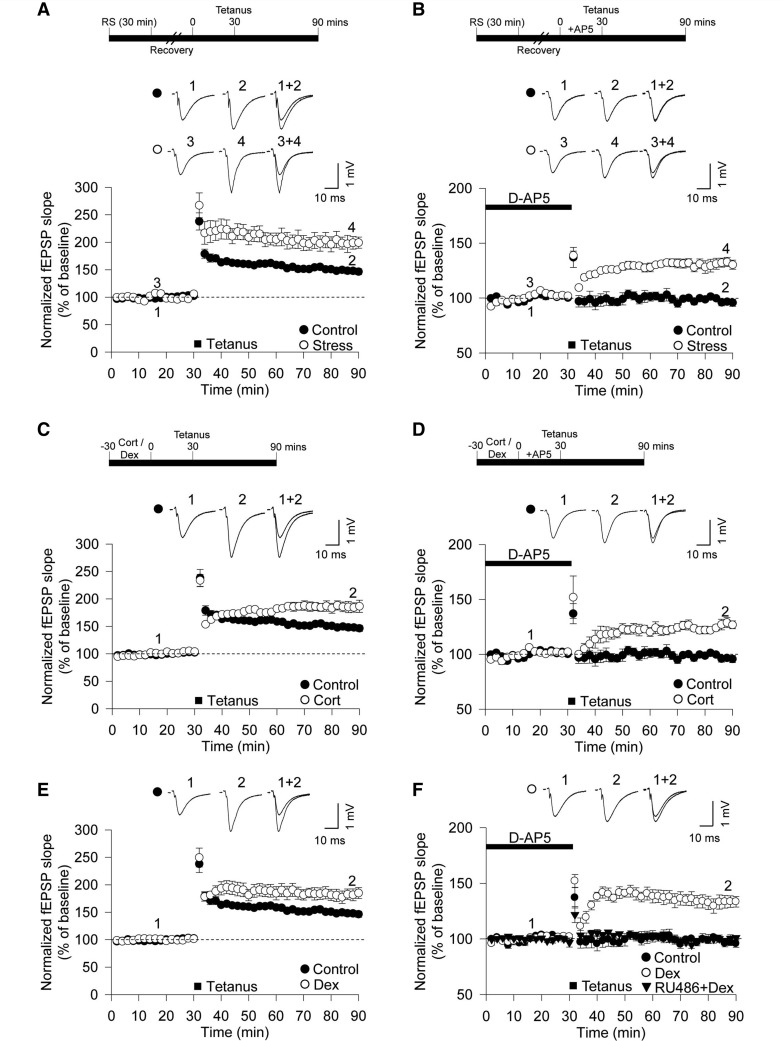
Brief restraint stress and glucocorticoid treatment facilitates LTP through an NMDA receptor-independent mechanism. (**A**) Delivery of high frequency stimulation (two trains of 100 Hz, 100 pulses) induced LTP in the CA1 of the hippocampus. Exposure to 30 min restraint stress (RS) increased the level of LTP (open circle, *n = *6) compared with non-stressed animals (closed circle; *n = *7). (**B**) Incubation with the NMDAR antagonist D-AP5 (50 µM), during high-frequency stimulation, completely abolished LTP in control rat slices (closed circle; *n = *6) but not after 30 min restraint stress (open circle, *n = *6). (**C**) Preincubation of slices with 200 nM corticosterone (Cort) facilitated LTP induction (*n = *6). (**D**) D-AP5 (50 µM) failed to block LTP in slices incubated with corticosterone (*n = *6). (**E**) Facilitation of LTP induction was observed after treatment with 200 nM dexamethasone (Dex; *n = *6). (**F**) D-AP5 (50 µM) failed to abolish LTP in slices treated with dexamethasone (*n = *6), but pretreatment with RU486 (500 nM) abolished the dexamethasone-mediated facilitation of LTP (*n = *6). Error bars indicate standard error of the mean (SEM). fEPSP = field excitatory postsynaptic potential.

To investigate the effects of acute stress on synaptic function, we examined hippocampal synaptic plasticity in slices taken from animals that had undergone brief restraint stress for 30 min immediately before being sacrificed. The magnitude of LTP in field excitatory postsynaptic potentials was significantly greater in stressed animals compared with control animals (stress: 199 ± 11% of baseline, *n = *6, open circle; interleaved controls: 150 ± 5%, *n = *7, closed circle, *P < *0.01, Fig. 1A). LTP induced by a tetanus (high-frequency stimulation, two trains of 100 Hz, 100 pulses) relies on the synaptic activation of NMDARs in the hippocampus (Collingridge *et al.*, 1983). However, the enhanced LTP induced by stress (stress-induced LTP) was independent of NMDAR activation, as the NMDAR antagonist D-AP5 completely blocked LTP in control slices (97 ± 3%, *n = *6, closed circle, *P < *0.01, Fig. 1B), but not in slices from stressed animals (132 ± 4%, *n = *6, open circle; Fig. 1B). Thus, acute stress enhances LTP through promoting an NMDAR-independent form of synaptic plasticity.

To determine whether the effects of stress on LTP were mediated by glucocorticoids, we performed experiments using corticosterone (200 nM) or the synthetic glucocorticoid receptor agonist dexamethasone (200 nM) applied for 30 min before the tetanus and continued until the end of the experiment (Fig. 1C and E). These treatments produced effects strikingly similar to acute stress (Fig. 1A and B). Both corticosterone and dexamethasone enhanced LTP (corticosterone: 185 ± 10%, *n = *6, Fig. 1C; dexamethasone: 182 ± 10%, *n = *6, Fig. 1E), and D-AP5 failed to eliminate LTP in corticosterone- and dexamethasone-treated slices (corticosterone: 129 ± 5%, *n = *6, Fig. 1D; dexamethasone: 133 ± 6%, *n = *6, Fig. 1F). Pretreatment with the glucocorticoid receptor antagonist RU486 (500 nM) completely abolished NMDAR-independent LTP, induced by dexamethasone treatment (99 ± 4%, *n = *6, Fig. 1F). In contrast, it had no effect on the induction of NMDAR-dependent LTP when applied alone (data not shown). Taken together, these results suggest that brief exposure to stress, acting through glucocorticoid receptors, enhances the magnitude of LTP in the hippocampus by priming an NMDAR-independent form of LTP.

**Figure 2:**
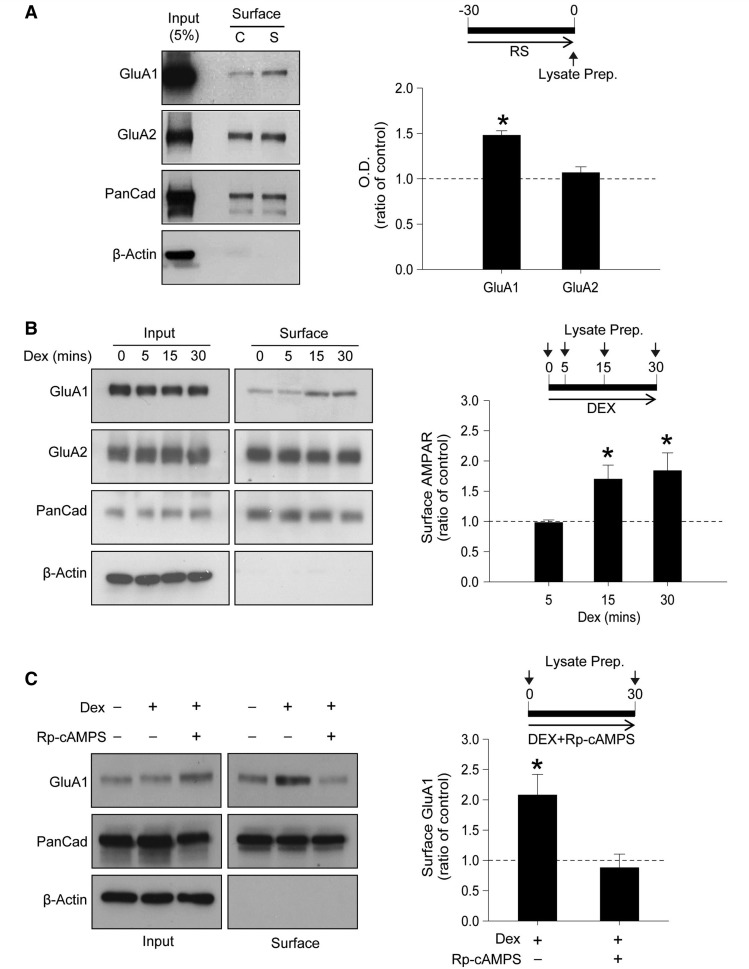
Increased surface expression of GluA1 following acute stress and dexamethasone treatment requires PKA activation. (**A**) Biotinylation assay using hippocampal slices from rats exposed to 30 min restraint stress. GluA1 surface expression is increased in the stressed animals (S) compared with non-stressed, control animals (C) (*n = *4). (**B**) *In vitro* experiments showing increased surface expression of GluA1 receptors after 15 and 30 min dexamethasone (Dex, 200 nM) treatment. No change in GluA2 expression was observed (*n = *3). (**C**) Pretreatment with 100 µM Rp-cAMPS completely abolished the increase in GluA1 surface expression following dexamethasone treatment (*n = *3). Error bars indicate SEM. *P < 0.05. PanCad = PanCadherin.

It is widely accepted that AMPARs mediate the major component of fast excitatory synaptic transmission (Derkach *et al.*, 2007; Kessels and Malinow, 2009) and that GluA2 lacking, homomeric GluA1 AMPARs can have an important role in the generation of LTP under some (Plant *et al.*, 2006; Wiltgen *et al.*, 2010; Tayler *et al.*, 2011), but not all (Adesnik and Nicoll, 2007), conditions. We therefore wondered whether the modulation of excitatory synaptic transmission by acute stress and glucocorticoid exposure, manifested in the enhancement of LTP, might be associated with changes in the synaptic expression of AMPARs and their subunit composition. Using a surface biotinylation assay, we found that there was a significant increase in surface expression of GluA1 in hippocampal slices prepared from stressed animals compared with controls (*P < *0.01, *n = *4, Fig. 2A). However, no difference was observed in GluA2 surface expression between the groups (*P* > 0.05, *n = *4, Fig. 2A).

Similar results were obtained in slices treated acutely with dexamethasone (Fig. 2B). In these experiments we performed surface biotinylation experiments at various times after adding dexamethasone to establish how rapidly the effect occurred. We observed no significant difference in AMPAR receptor expression after 5 min (*P > *0.05, *n = *3) but GluA1 surface expression was selectively increased at 15 and 30 min (*P < *0.05, *n = *3 for both time-points, Fig. 2B). Given that there was no change in the expression of the GluA2 subunit, these results suggest that acute exposure to dexamethasone may increase the surface expression of homomeric GluA1-containing AMPARs. The surface expression of GluA1 AMPARs is known to be regulated by a PKA-dependent signalling mechanism (Lee *et al.*, 2003; Lu and Roche, 2011). Therefore, we tested whether pretreatment with the PKA inhibitor Rp-cAMPS would affect the dexamethasone-mediated enhancement of GluA1 surface expression. Pretreating slices with Rp-cAMPS (100 μM) abolished the effect of dexamethasone (*P < *0.05, *n = *3, Fig. 2C), implicating PKA in this glucocorticoid-mediated regulation of AMPAR surface expression.

### Acute stress and glucocorticoids increase S845 phosphorylation of GluA1

**Figure 3:**
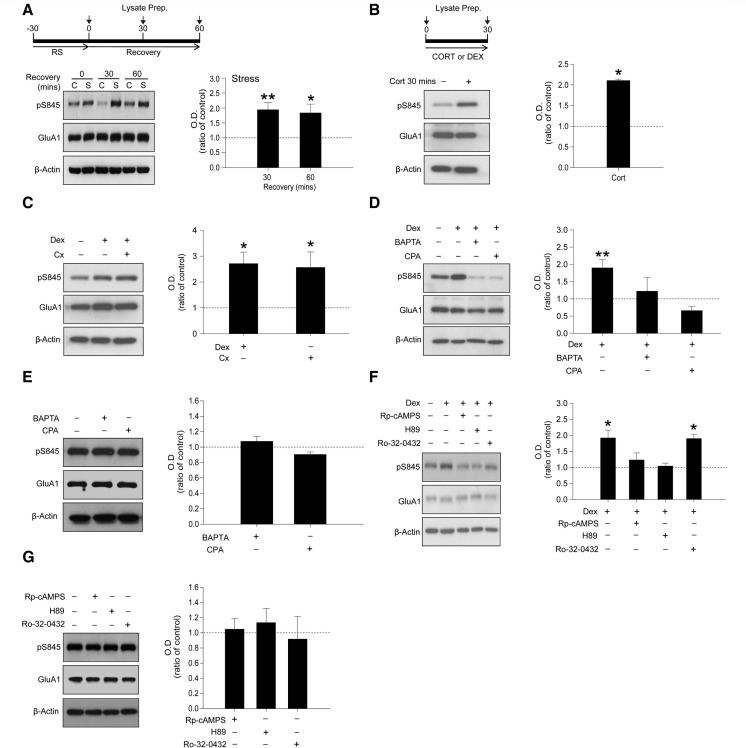
Glucocorticoids enhance the phosphorylation of GluA1 via a non-genomic mechanism requiring increased Ca^2+^ mobilization. (**A**) Animals were exposed to 30 min restraint stress. Hippocampal slices were homogenized immediately following stress or allowed to recover for either 30 or 60 min. Phosphorylation levels of serine 845 (pS845) of the GluA1 sub-unit of AMPARs were increased in the stressed animals (S) compared to non-stressed control animals (C) (*n = *5). (**B**) Preincubation with corticosterone (Cort, 200 nM) increased pS845 of GluA1 compared to control brain slices (*n = *3). (**C**) Pretreatment with cycloheximide (cx; 100 µM) had no effect on pS845 levels following dexamethasone (Dex) treatment (*n = *5). (**D**) Both BAPTA-AM (100 μM) and cyclopiazonic acid (CPA, 50 μM) preincubation prevented the increased phosphorylation of the GluA1 subunits induced by dexamethasone treatment (*n = *4). (**E**) Treatment with BAPTA-AM and cyclopiazonic acid alone had no effect on pS845 (*n = *4). (**F**) Pretreatment with PKA inhibitors Rp-cAMPS (100 µM) or H89 (10 µM) abolished dexamethasone-mediated increases in pS845 levels, whereas the protein kinase C inhibitor Ro-32-0432 (10 µM) had no effect (*n = *4). Error bars indicate SEM. *P < 0.05; **P < 0.01. (**G**) Treatment with Rp-cAMPS, H89 or Ro-32-0432 alone had no effect on pS845 levels (*n = *4).

The PKA-mediated phosphorylation of the serine 845 (pS845) residue of the GluA1 subunit correlates with the surface expression of homomeric GluA1 AMPARs on the plasma membrane (Mammen *et al.*, 1997; Lee *et al.*, 2003; Oh *et al.*, 2006). Western blot analysis of *ex vivo* samples indicated that stress induced a significant increase in pS845 that persisted for at least 60 min after slice preparation (*P < *0.05, *n = *5, Fig. 3A). Consistent with the *ex vivo* experiments, incubation for 30 min with either corticosterone (*P < *0.05, *n = *3, Fig. 3B) or dexamethasone (*P < *0.05, *n = *5, Fig. 3C) induced comparable increases in pS845. As glucocorticoid receptor activation was found to be important in the induction of stress-induced LTP (Fig. 1F), we also determined whether the glucocorticoid receptor was an important mediator in pS845 following dexamethasone treatment. The increase in pS845 following dexamethasone treatment (*P < *0.05, *n = *4, Supplementary Fig. 1A) was abolished by pretreatment with RU486 (500 nM), indicating an important role for glucocorticoid receptor in this acute stress model.

Many well-characterized effects of stress involve alterations in gene transcription and translation (Bain *et al.*, 2007; Heitzer *et al.*, 2007) although fast, non-genomic actions of glucocorticoids are also documented (Losel and Wehling, 2003; Dallman, 2005). We examined whether the pS845 required *de novo* protein synthesis by using the translation inhibitor cycloheximide. Pretreatment for 30 min with 100 µM cycloheximide had no effect on the ability of dexamethasone (30 min) to increase the phosphorylation of S845 (*P > *0.05, *n = *5, Fig. 3C), suggesting that a non-genomic mechanism is involved in this action.

As Ca^2+^ is important for most, but not all, forms of synaptic plasticity (Esteban *et al.*, 2003), we next tested whether glucocorticoid-mediated pS845 of GluA1 requires Ca^2+^. To test this, we modified levels of Ca^2+^ using the membrane-permeable Ca^2+^ chelator 1,2-bis(2-aminophenoxy)ethane-*N*,*N*,*N*',*N*'-tetraacetic acid tetrakis (acetoxymethyl ester) (BAPTA-AM). Dexamethasone application alone significantly increased pS845 under control conditions but not following 30 min pretreatment with BAPTA-AM, (dexamethasone versus dexamethasone + BAPTA: *P < *0.05, *n = *4, Fig. 3D). A primary source of intracellular Ca^2+^ involved in certain forms of LTP is Ca^2+^ released from intracellular stores (Rose and Konnerth, 2001). To investigate its possible involvement in the effects of dexamethasone we used the sarcoplasmic Ca^2+^-ATPase inhibitor cyclopiazonic acid, as a means of depleting intracellular stores of Ca^2+^. Pretreatment for 30 min with 50 μM cyclopiazonic acid completely abolished the effects of dexamethasone treatment on pS845 (dexamethasone versus dexamethasone +cyclopiazonic acid: *P < *0.05, *n = *4, Fig. 3D). Moreover, BAPTA-AM and cyclopiazonic acid treatment alone had no significant effect on pS845 (*P > *0.05 for all treatments, *n = *4, Fig. 3E). These data suggest that intracellular Ca^2+^ mobilization is important for glucocorticoid-induced S845 phosphorylation.

Given the role of PKA in the dexamethasone-induced increase in surface GluA1 (Fig. 2) and in the phosphorylation of S845 (Banke *et al.*, 2000), it seemed likely that this kinase mediates the increased pS845 in response to glucocorticoids. Consistent with this, preincubation for 30 min with the PKA inhibitors Rp-cAMPS (100 µM) or H89 (10 µM), prevented the effects of dexamethasone on pS845 (*P < *0.05 for all treatments, *n = *4, Fig. 3F). In contrast, the protein kinase C inhibitor tested, Ro-32-0432 (10 µM) was without effect (*P > *0.05, *n = *4, Fig. 3F). Furthermore, treatment with PKA and the protein kinase C inhibitor alone produced no significant change in pS845 (*P > *0.05 for all treatments, *n = *4, Fig. 3G). Collectively, these results indicate that acute stress, via the mobilization of intracellular Ca^2+^, induces PKA activation and leads to the insertion of GluA1, but not GluA2, subunits into the plasma membrane.

**Figure 4:**
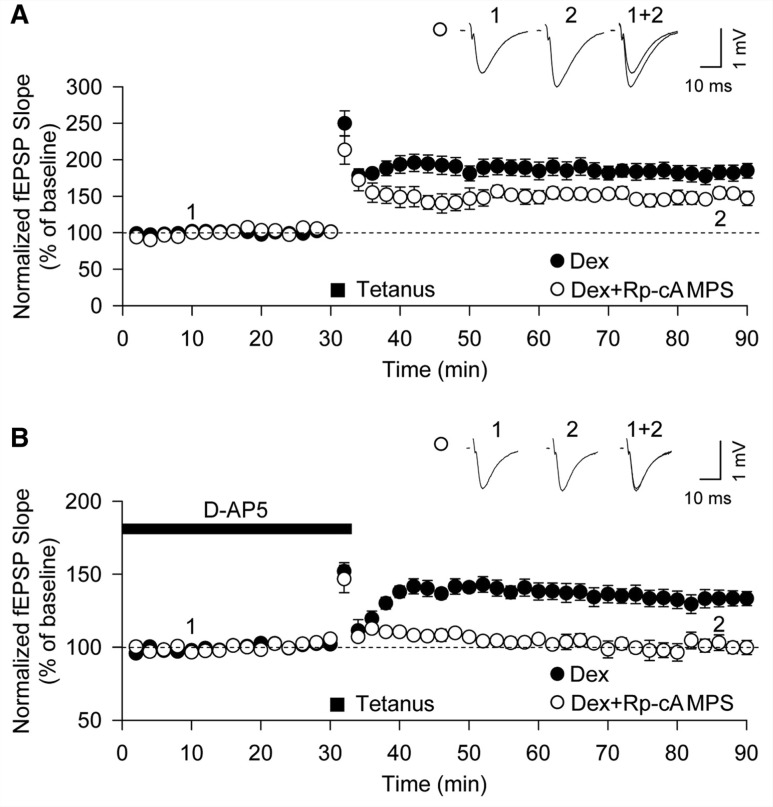
Stress-induced LTP requires PKA activation. (**A**) Enhancement of LTP by dexamethasone (Dex) treatment (closed circle; *n = *6) was attenuated by pretreatment with 100 µM Rp-cAMPS (open circle; *n = *5). (**B**) Rp-cAMPS (100 µM) blocked stress-induced LTP induced in the presence of D-AP5 (open circle; *n = *5). fEPSP = field excitatory postsynaptic potential.

The PKA-dependent actions of dexamethasone upon pS845 and surface expression of GluA1 subunits may be causally related to the generation of stress-induced LTP or may be an epiphenomenon. To distinguish between these possibilities, we examined whether Rp-cAMPS affected the dexamethasone-induced enhancement of LTP. Preincubation with Rp-cAMPS resulted in the inhibition of enhanced LTP in the presence of dexamethasone (from 182 ± 10%, *n = *6 to 155 ± 9%, *n = *5; *P < *0.05, Fig. 4A), a value not significantly different to that seen in control slices. Furthermore, Rp-cAMPS eliminated stress-induced LTP induced in the presence of D-AP5 following treatment with dexamethasone (101 ± 4%, *n = *5, Fig. 4B). These findings are most readily explained by a process whereby the activation of glucocorticoid receptors increase GluA1-AMPAR surface expression through PKA signalling, and this permits the induction of an NMDAR-independent LTP that is additive to NMDAR-dependent LTP.

### Stress-induced long-term potentiation is triggered by Ca^2+^-permeable AMPA receptors

**Figure 5:**
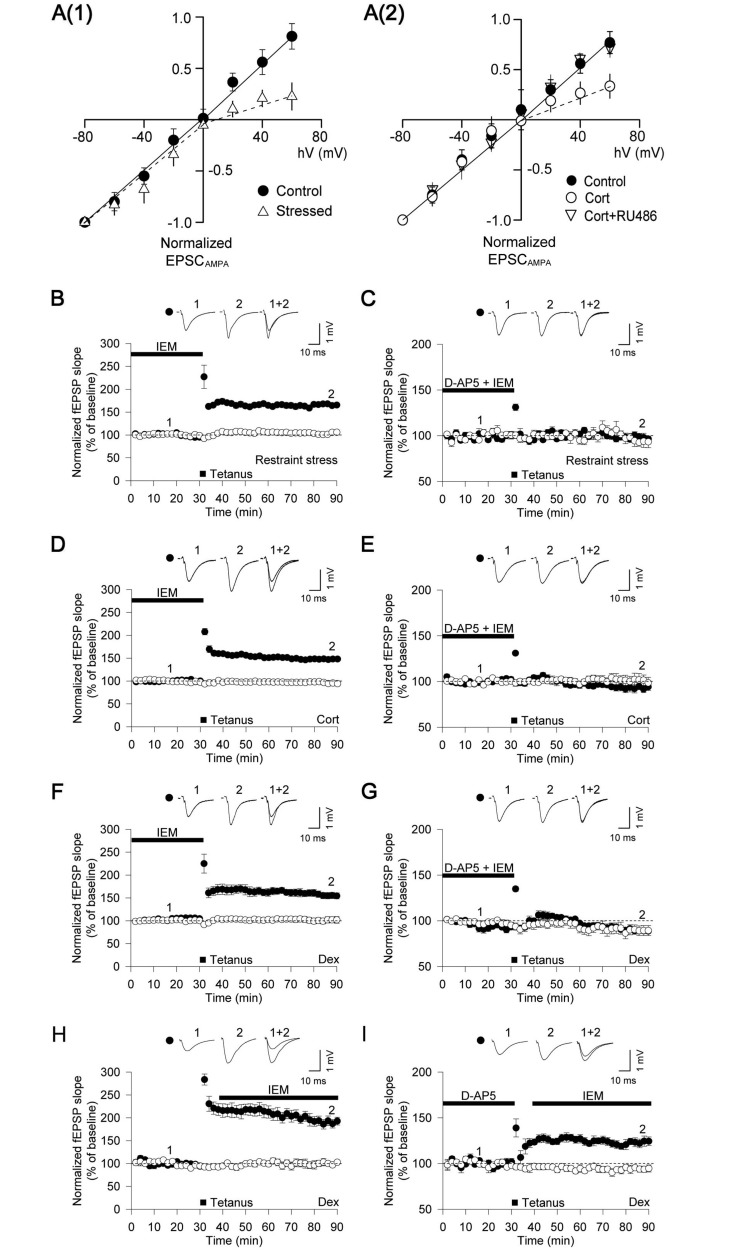
Stress and glucocorticoids facilitate LTP through a mechanism involving Ca^2+^-permeable AMPARs. (**A1**) Acute restraint stress induces an inwardly rectified I-V relationship of AMPAR current (EPSC_AMPA_) (control, *n = *9; stressed, *n = *7). Spermine (100 µM) was included in the filling solution and EPSC_AMPA_ was isolated by applying the NMDAR antagonists D-AP5 (50 µM) and MK801 (10 µM). (**A2**) Corticosterone (Cort) treatment induces an inwardly rectified I-V relationship of EPSC_AMPA_. This is attenuated by pretreatment with RU486 (500 nM) in acute slices (control, *n = *7; corticosterone, *n = *7; corticosterone + RU486, *n = *7). (**B–I**) Filled symbol indicates tetanus delivered input and open symbol indicates control input. (**B**) The Ca^2+^-permeable AMPAR antagonist IEM-1460 (IEM: 100 µM) attenuated facilitation of LTP recorded following restraint stress (*n = *6). (**C**) IEM abolished stress-induced LTP (tetanus delivered in presence of D-AP5), induced by restraint stress (*n = *6). (**D**) IEM attenuated LTP recorded following corticosterone treatment (*n = *6). (**E**) IEM abolished stress-induced LTP (tetanus delivered in presence of D-AP5), induced by corticosterone treatment (*n = *6). (**F**) IEM attenuated LTP recorded after dexamethasone treatment (*n = *6). (**G**) IEM abolished stress-induced LTP (tetanus delivered in presence of D-AP5), induced by dexamethasone treatment (*n = *6). (**H**) IEM had no effect on pre-established LTP induced by dexamethasone treatment (*n = *7). (**I**) IEM had no effect on the expression of stress-induced LTP (tetanus delivered in presence of D-AP5), enabled by dexamethasone treatment (*n = *7). EPSP = excitatory postsynaptic potential; fEPSP = field excitatory postsynaptic potential.

CP-AMPARs have been shown to mediate the induction of an NMDAR-independent form of LTP at CA1 in the hippocampus of mice lacking GluA2 AMPARs (Asrar *et al.*, 2009). As we had observed that stimulation of glucocorticoid receptors results in an increase in surface GluA1, it seemed plausible that stress-induced LTP is also triggered via CP-AMPARs. To confirm this hypothesis we examined the synaptic current-voltage (I-V) relationship of excitatory postsynaptic currents (EPSC)_AMPA_. As expected, the I-V relationship was linear in control animals. In contrast, the restraint stress rats exhibited an inwardly rectifying I-V curve [*P < *0.01, *n = *7, Fig. 5A(i)]. A similarly rectified I-V curve was observed in slices treated with corticosterone [*P < *0.01, *n = *7, Fig. 5A(ii)]. This effect could be blocked with pretreatment with RU486 [*n = *7, Fig. 5A(ii)]. This inwardly rectified I-V relationship is suggestive of an increase in the synaptic expression of CP-AMPARs. To further clarify the role of CP-AMPARs in stress-induced LTP we tested whether the CP-AMPAR inhibitor IEM (100 µM) (Buldakova *et al.*, 2007; Asrar *et al.*, 2009) affected LTP in *ex vivo* hippocampal slices prepared from stressed rats. The level of LTP observed after application of IEM (168 ± 4%, *n = *6, Fig. 5B) was less than that obtained from untreated slices from stressed rats (199 ± 11%, *n = *6; *P < *0.01, Fig. 1A) but similar to that observed in control rats (150 ± 5%, *n = *7; *P > *0.05, Fig. 1A), suggesting an inhibition of the stress-induced LTP component. Consistent with this, IEM completely prevented the induction of NMDAR-independent LTP in slices from stressed rats (99 ± 3%, *n = *6, Fig. 5C). We next performed experiments using acute slices treated with either corticosterone or dexamethasone. Consistent with the *ex vivo* experiments from stressed rats, facilitation of LTP by corticosterone or dexamethasone was prevented by IEM (corticosterone: 148 ± 3%, *n = *6, Fig. 5D; dexamethasone: 155 ± 7%, *n = *6, Fig. 5F). Furthermore, IEM completely blocked LTP, induced by glucocorticoid receptor stimulation in the presence of D-AP5 (corticosterone: 95 ± 4%, *n = *6, Fig. 5E; dexamethasone: 91 ± 2%, *n = *6, Fig. 5G).

These data indicate that CP-AMPAR are involved in the induction of an NMDAR-independent form of LTP. In some (Plant *et al.*, 2006) but not all (Adesnik and Nicoll, 2007) circumstances, the insertion of CP-AMPARs may also contribute to the early expression of LTP. To determine whether this is the case for stress-induced LTP, we applied IEM to slices after high-frequency stimulation. IEM had no effect on the dexamethasone-enhanced LTP under either control conditions (186 ± 10%, *n = *7, Fig. 5H), or in the presence of D-AP5 (125 ± 6%, *n = *7, Fig. 5I). As the magnitude of stress-induced LTP was not affected by IEM post-tetanus, we concluded that CP-AMPARs are specifically involved during the induction of stress-induced LTP. These results therefore provide a physiological role for CP-AMPARs in the induction of a form of LTP in response to acute stress.

### Stress-induced long-term potentiation is primed by the activation of NMDA receptors

**Figure 6:**
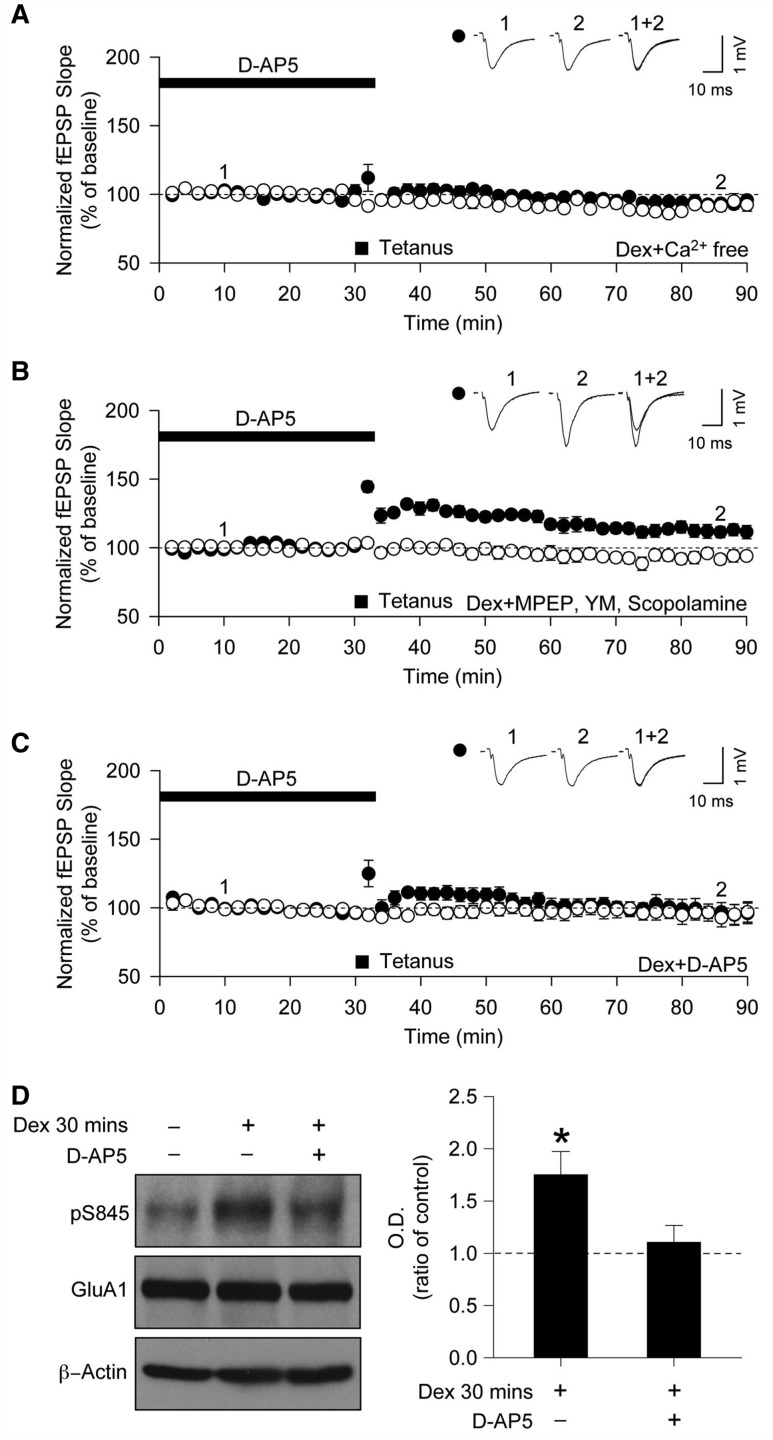
Ca^2+^-influx through NMDARs is required for dexamethasone to generate stress-induced LTP. (**A–C**) Filled symbol indicates tetanus delivered input and open symbol indicates control input. (**A**) Incubation with a Ca^2+^-free medium during dexamethasone (Dex) treatment prevented stress-induced LTP (*n = *6). (**B**) Incubation with MPEP, YM-298198 and scopolamine during dexamethasone treatment did not affect the generation of stress-induced LTP (*n = *6). (**C**) Incubation with D-AP5 during dexamethasone treatment prevented stress-induced LTP (*n = *6). (**D**) Incubation with D-AP5 abolished dexamethasone-mediated enhancement of pS845 (*n = *4). fEPSP = field excitatory postsynaptic potential. *P < 0.05.

A key question concerns how the stimulation of glucocorticoid receptors leads to the state where a tetanus can induce an NMDAR-independent form of LTP; how do glucocorticoids recruit CP-AMPARs to prime the synapses for stress-induced LTP? The finding that the phosphorylation of pS845 requires Ca^2+^ suggests that the priming mechanism likely involves a change in intracellular Ca^2+^. To test for this, we used a Ca^2+^-free artificial CSF buffer during the dexamethasone treatment period and then reintroduced Ca^2+^ to test for the presence of NMDAR-independent LTP. Stress-induced LTP was absent in dexamethasone-treated slices that had been incubated in Ca^2+^-free artificial CSF (96 ± 3%, *n = *6, Fig. 6A). This suggests that Ca^2+^ is required for the priming mechanism by which glucocorticoids recruit CP-AMPARs to enable stress-induced LTP.

Ca^2+^ can be elevated in neurons through various pathways, including the activation of G-coupled receptors. The predominant G_q/11_-coupled receptors in CA1 neurons are mGlu_1_, mGlu_5_ and m1AChR, all of whose activation can induce release Ca^2+^ from intracellular stores. We found, however, that a cocktail of inhibitors for these receptors (2-methyl-6-(phenylethynyl)pyridine hydrochloride) (MPEP 1 µM; mGluR5 antagonist), desmethyl-YM-298198 (YM; 500 nM; mGluR1 antagonist) and scopolamine (20 µM; mAChR antagonist) could not prevent stress-induced LTP (115 ± 4%, *n = *6, Fig. 6B). The synaptic activation of postsynaptic NMDARs also regulates Ca^2+^ mobilization (Alford *et al.*, 1993; Yuste *et al.*, 2000). We therefore tested whether Ca^2+^ flux through NMDARs is involved in the priming of stress-induced LTP. Interestingly, LTP was completely blocked by D-AP5 in slices pretreated with dexamethasone plus D-AP5 (96 ± 7%, *n = *6, Fig. 6C). Consistent with these data, we found that 30 min dexamethasone treatment caused an increase in pS845 (*P < *0.05, *n = *4, Fig. 6D), which was prevented by pretreatment with D-AP5. These results suggest that the induction of stress-induced LTP requires the glucocorticoid-induced activation of NMDARs and subsequent Ca^2+^ influx.

## Discussion

Alterations in synaptic function after either acute stress or emotional arousal can facilitate cognition (Cahill *et al.*, 1994; Shors and Servatius, 1995; Shors and Mathew, 1998; Blank *et al.*, 2002; Weiss *et al.*, 2005; Joels *et al.*, 2006; Bangasser and Shors, 2007).This enhanced cognitive performance after exposure to a stressor serves an important survival strategy, and understanding the underlying mechanism could provide insights into new translational strategies for the treatment of memory impairment in man. The first step is to ascertain the underlying mechanism, and we now describe a novel mechanism by which acute stress can enhance synaptic plasticity, a major process involved in learning and memory (Bliss and Collingridge, 1993). We find that stimulation of glucocorticoid receptors leads to an NMDAR-dependent form of metaplasticity that involves the PKA-dependent insertion of GluA1-containing CP-AMPARs into synapses. These newly inserted AMPARs can then induce a form of LTP that is entirely independent of NMDARs but that is additive to conventional NMDAR-dependent LTP. In this way, acute stress is able to enhance the magnitude of LTP at hippocampal synapses (Supplementary Fig. 1B).

Previous studies have shown that glucocorticoids are able to rapidly modify glutamatergic function. For example, it has been shown that corticosterone induces the mobilization of GluA1 and GluA2 to the plasma membrane and can facilitate a chemical form of LTP observed in dissociated hippocampal neurons (Groc *et al.*, 2008; Conboy and Sandi, 2009). The effect is complex, with time dependent actions through muscarinic receptors and glucocorticoid receptors (Groc *et al.*, 2008; Yuen *et al.*, 2011). In prefrontal cortex neurons, acute stress and corticosterone have been shown to enhance synaptic transmission, through facilitated AMPAR and NMDAR function (Yuen *et al.*, 2009, 2011; Lee *et al.*, 2012). More broadly, acute stress or exposure to glucocorticoids can cause a rapid increase in glutamate release (Moghaddam, 1993; Abraham *et al.*, 1996; Venero and Borrell, 1999; Reznikov *et al.*, 2007). Although these changes in excitatory synaptic transmission have been suggested to regulate synaptic plasticity, no underlying rapid non-genomic mechanism has been revealed. Our study has shown a role for the selective insertion of CP-AMPARs that enables the induction of an NMDAR-independent form of LTP.

AMPARs that lack the GluA2 subunit, such as homomeric GluA1 receptors, have a higher Ca^2+^ permeability and single channel conductance than GluA2 containing AMPARs (Lomeli *et al.*, 1994; Swanson *et al.*, 1997). These properties have been shown to be important for the induction and/or expression of various forms of synaptic plasticity in the CNS (Mahanty and Sah, 1998; Liu and Cull-Candy, 2000; Lei and McBain, 2002; Cull-Candy *et al.*, 2006; Liu and Zukin, 2007; Shepherd, 2012). For example, it has been shown that in transgenic mice lacking GluA2 that CP-AMPARs can mediate a form of NMDAR-independent LTP (Jia *et al.*, 1996). In addition, it has been shown that CP-AMPARs can be transiently inserted following the induction of NMDAR-LTP, where they trigger the subsequent expression of neuronal plasticity (Plant *et al.*, 2006; but see also Adesnik and Nicoll, 2007). We found that in stress-induced LTP, CP-AMPARs are required for the induction, rather than the expression, of LTP. Thus, IEM was able to fully block the induction of NMDAR-independent LTP as observed either in isolation from or in addition to NMDAR-LTP. However, IEM had no effect on either baseline transmission or the expression of LTP when applied shortly after its induction. Further work will be required to establish the precise mechanism of stress-induced LTP expression and how this is initiated through the transient activation of CP-AMPARs. One possible explanation is that stress results in the insertion of CP-AMPARs into the plasma membrane at extra-synaptic sites. During high-frequency stimulation these could become activated, possibly by the ‘spill-over’ of l-glutamate (Yang *et al.*, 2005; Okubo *et al.*, 2010), triggering the synaptic insertion of GluA2-containing AMPARs.

Although NMDARs are not required for the induction of stress-induced LTP, their activation is essential for the metaplasticity that primes CA1 synapses for stress-induced LTP. Thus, inhibition of NMDARs during the application of dexamethasone completely prevented the subsequent ability to induce NMDAR-independent LTP. This leads to the question regarding the mechanism underlying this important form of metaplasticity. It is known that glucocorticoids can rapidly enhance NMDAR activation and subsequently increase intracellular Ca^2+^ levels in the CA1 region of the hippocampus (Takahashi *et al.*, 2002; Sato *et al.*, 2004; Xiao *et al.*, 2010). Our observation that this form of metaplasticity is prevented by BAPTA is consistent with these observations. The synaptic activation of NMDARs is known to result in the release of Ca^2+^ from intracellular stores (Alford *et al.*, 1993) and we have found that release from stores is required for the metaplasticity. This priming is specific to NMDAR activation since inhibition of G-protein coupled receptors, which can also modulate the release of Ca^2+^ from intracellular stores, did not affect stress-induced LTP. We also found that PKA is required for the priming of stress-induced LTP, and that its activation is associated with pS845 and the increased surface expression of GluA1 subunits. The most plausible mechanism therefore is that Ca^2+^ associated with NMDAR stimulation activates a Ca^2+^-sensitive adenylyl cyclase (Pierre *et al.*, 2009), which leads to PKA-mediated phosphorylation of S845 to trigger the AMPAR trafficking that underlies the priming effect. Consistent with this model, NMDAR triggered activation of PKA (Roberson and Sweatt, 1996) has been shown to drive the synaptic expression of GluA1-containing AMPARs (Esteban *et al.*, 2003). This mechanism does not require *de novo* protein synthesis and accordingly, we found that the ability of dexamethasone to prime stress-induced LTP was unaffected by treatment with cycloheximide. However, the possibility remains that stress could have a secondary action to upregulate the gene expression of components of this pathway to achieve a longer lasting effect. In this regard, it is interesting to note that PKA has also been shown to play a role in a late, protein synthesis-dependent phase of LTP (Frey *et al.*, 1993; Huang and Kandel, 1994; Abel *et al.*, 1997; Nayak *et al.*, 1998).

There is strong evidence to support the notion that acute stress can facilitate memory through enhanced synaptic plasticity (Conrad *et al.*, 1999; Blank *et al.*, 2002; Hui *et al.*, 2004; Nijholt *et al.*, 2004; Yuen *et al.*, 2011), though the mechanism underlying this process is unknown. To establish how this mechanism contributes to cognition is a major undertaking, given how much time has been devoted to understanding how NMDAR-dependent LTP is involved in learning and memory (Morris *et al.*, 1986; Martin *et al.*, 2000; Neves *et al.*, 2008); a topic for which aspects of the relationship still remain controversial (Bannerman *et al.*, 2012). It is interesting to note, however, that in animals engineered to lack GluA2, the total level of LTP and the proportion that is dependent on NMDARs is similar to those observed in this present study after acute stress (Jia *et al.*, 1996). Studies have shown that NMDAR-dependent and independent forms of LTP mediate different behaviours (Wiltgen *et al.*, 2010). It has also been proposed that NMDAR-independent LTP might explain the resistance of hippocampal-dependent learning to NMDAR antagonism observed in water maze tasks (Morris *et al.*, 1986; Bannerman *et al.*, 1995, 2012) and other behaviours (Abel *et al.*, 1997) under certain conditions, most notably in the ‘upstairs/downstairs’ water maze experiments (Morris *et al.*, 1986; Bannerman *et al.*, 1995). However, no physiological context has previously been found where this occurs in normal animals. Therefore, our findings that acute stress readily induces an NMDAR-independent form of LTP may be especially pertinent in this context (Wiltgen *et al.*, 2010).

Therefore, it is plausible that the priming of synaptic plasticity observed in this study may be associated with periods of heightened cognition. As modifications in synaptic function are thought to be fundamental in the efficient formation of memory (Alford *et al.*, 1993; Blank *et al.*, 2002; Pierre *et al.*, 2009), we propose that glucocorticoids play a role in fine-tuning synaptic function and regulating the memory trace through the expression of CP-AMPARs.

## Supplementary Material

Supplementary Data

## Abbreviations

AMPAR: α-amino-3-hydroxy-5-methylisoxazole-4-propionic acid receptor
BAPTA-AM: 1,2-bis(2-aminophenoxy)ethane-*N*,*N*,*N*',*N*'-tetraacetic acid tetrakis (acetoxymethyl ester)
LTP: long-term potentiation
NMDAR: *N*-methyl-d-aspartic acid receptor
PKA: protein kinase A
