# Supplementary material for: Acute stress causes rapid synaptic insertion of Ca2+-permeable AMPA receptors to facilitate long-term potentiation in the hippocampus
Source: Brain. 2013 Dec 10;136(12):3753–65. doi: 10.1093/brain/awt293 (PMC3859225; doi:10.1093/brain/awt293)
Supplement: Supplementary Data [file supp_136_12_3753__index.html]

Acute stress causes rapid synaptic insertion of Ca2+-permeable AMPA receptors to facilitate long-term potentiation in the hippocampus — Acute stress causes rapid synaptic insertion of Ca2+-permeable AMPA receptors to facilitate long-term potentiation in the hippocampus — Supplementary Data 

# Acute stress causes rapid synaptic insertion of Ca2+-permeable AMPA receptors to facilitate long-term potentiation in the hippocampus

## Supplementary Data

files

**Files in this Data Supplement:**

- Supplementary Data - jpg file
- Supplementary Data - docx file
