# Supplementary figures and images for: Acute stress causes rapid synaptic insertion of Ca2+-permeable AMPA receptors to facilitate long-term potentiation in the hippocampus
Source: Brain. 2013 Dec 10;136(12):3753–65. doi: 10.1093/brain/awt293 (PMC3859225; doi:10.1093/brain/awt293)

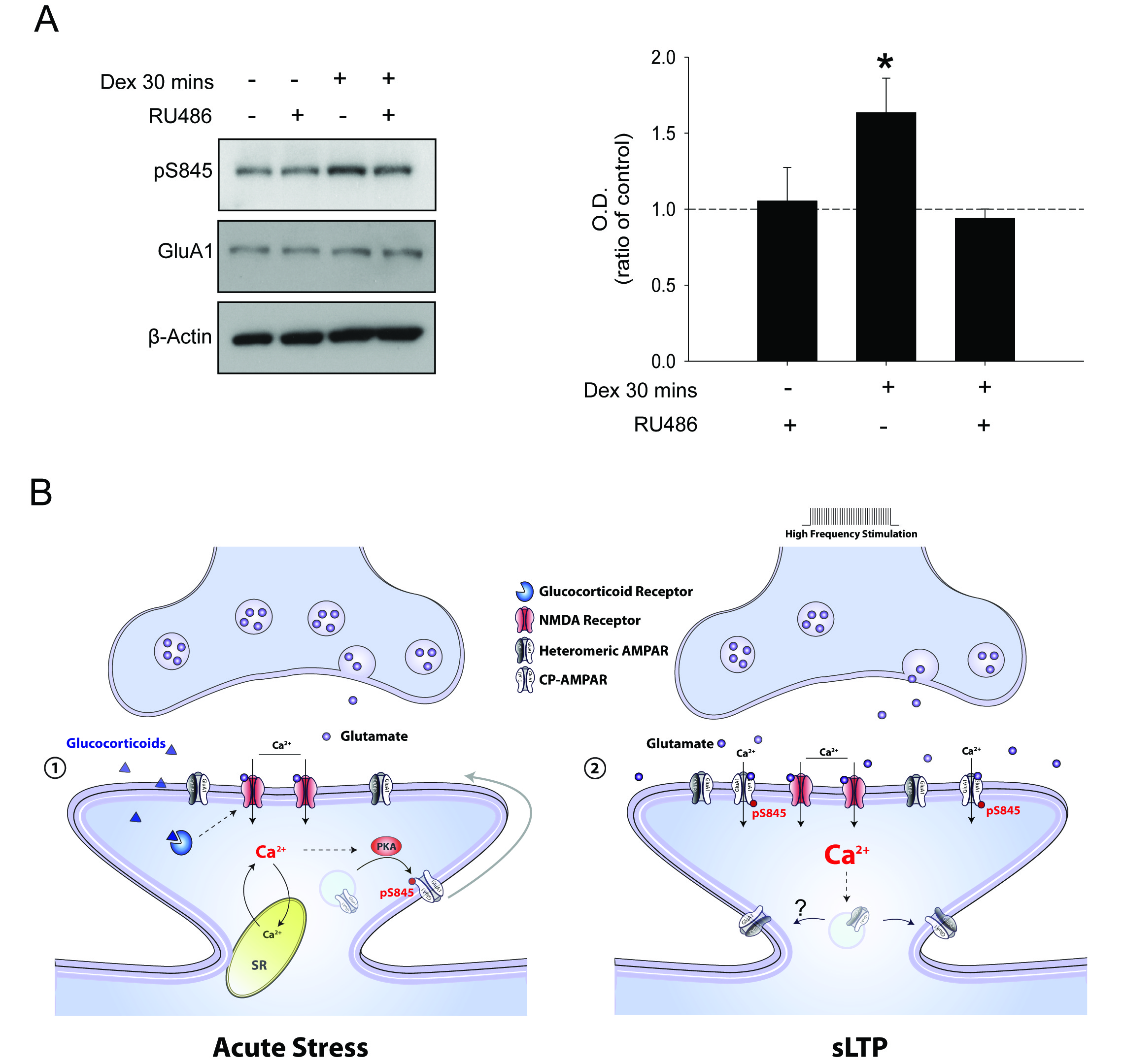

Supplement: Supplementary Data [file supp_awt293_brain-2013-00205-File008.jpg]
