# Supplementary material for: Acute stress causes rapid synaptic insertion of Ca2+-permeable AMPA receptors to facilitate long-term potentiation in the hippocampus
Source: Brain. 2013 Dec 10;136(12):3753–65. doi: 10.1093/brain/awt293 (PMC3859225; doi:10.1093/brain/awt293)
Supplement: Supplementary Data [file supp_awt293_brain-2013-00205-File009.docx]

**Supplementary Figure Legend**.

(**A**) Dex-mediated increase in pS845 is abolished by pre-treatment with 500 nM RU486 (*n* = 4).

(**B**) Schematic detailing how glucocorticoids regulate CP-AMPAR and induce sLTP in the hippocampus.
